# Computational prediction of the Crc regulon identifies genus-wide and species-specific targets of catabolite repression control in *Pseudomonas *bacteria

**DOI:** 10.1186/1471-2180-10-300

**Published:** 2010-11-25

**Authors:** Patrick Browne, Matthieu Barret, Fergal O'Gara, John P Morrissey

**Affiliations:** 1 BIOMERIT Research Centre, Microbiology Department University College Cork, Cork, Ireland; 2 Microbiology Department University College Cork, Cork, Ireland

## Abstract

**Background:**

Catabolite repression control (CRC) is an important global control system in *Pseudomonas *that fine tunes metabolism in order optimise growth and metabolism in a range of different environments. The mechanism of CRC in *Pseudomonas *spp. centres on the binding of a protein, Crc, to an A-rich motif on the 5' end of an mRNA resulting in translational down-regulation of target genes. Despite the identification of several Crc targets in *Pseudomonas *spp. the Crc regulon has remained largely unexplored.

**Results:**

In order to predict direct targets of Crc, we used a bioinformatics approach based on detection of A-rich motifs near the initiation of translation of all protein-encoding genes in twelve fully sequenced *Pseudomonas *genomes. As expected, our data predict that genes related to the utilisation of less preferred nutrients, such as some carbohydrates, nitrogen sources and aromatic carbon compounds are targets of Crc. A general trend in this analysis is that the regulation of transporters is conserved across species whereas regulation of specific enzymatic steps or transcriptional activators are often conserved only within a species. Interestingly, some nucleoid associated proteins (NAPs) such as HU and IHF are predicted to be regulated by Crc. This finding indicates a possible role of Crc in indirect control over a subset of genes that depend on the DNA bending properties of NAPs for expression or repression. Finally, some virulence traits such as alginate and rhamnolipid production also appear to be regulated by Crc, which links nutritional status cues with the regulation of virulence traits.

**Conclusions:**

Catabolite repression control regulates a broad spectrum of genes in *Pseudomonas*. Some targets are genus-wide and are typically related to central metabolism, whereas other targets are species-specific, or even unique to particular strains. Further study of these novel targets will enhance our understanding of how *Pseudomonas *bacteria integrate nutritional status cues with the regulation of traits that are of ecological, industrial and clinical importance.

## Background

The genus *Pseudomonas *is an important group of microorganisms that occupy a wide variety of habitats including soil [1], the rhizosphere [2], food [3] and mammalian hosts [4]. Some species are important plant or human pathogens, whereas others are involved in processes such as bioremediation [5], biocontrol [6-8], nutrient cycling [9] or biotechnological processes [10]. A key aspect of the lifestyle of Pseudomonads is their ability to adapt, grow and compete in a wide variety of habitats. Thus, Pseudomonads require great flexibility in controlling their diverse array of metabolic pathways and, like most microorganisms, have global regulatory systems that ensure that the best nutrient source is utilised and almost depleted before less favoured nutrient sources are exploited [11-13].

Pseudomonads favour the utilisation of organic acids, particularly tricarboxylic acid (TCA) cycle intermediates, and amino acids over various other carbon sources such as carbohydrates or hydrocarbons [14]. This is in contrast to the majority of well-studied *Enterobacteriaceae *and *Firmicutes*, which favour glucose and use a system known as carbon catabolite repression (CCR) or catabolite repression control (CRC) to regulate carbon utilisation. The mechanism of CCR in *Enterobacteriaceae *and *Firmicutes *centres on a protein phosphorylation cascade and also involves transcriptional regulation mediated through cyclic AMP (cAMP) binding to the cAMP receptor protein (Crp) (for review see [11,12]). Although Pseudomonads possess a Crp homolog, Vfr, this protein is not involved in carbon source regulation, at least in *P. aeruginosa *PAO1 [15]. In fact, the CRC mechanism used by Pseudomonads to regulate carbon source utilisation is fundamentally different to CCR of *Enterobacteriaceae *and *Firmicutes*.

A central mediator of CRC is the Crc protein, which acts as a post-transcriptional regulator of target genes [16]. The post-transcriptional action of Crc relies on the binding of Crc to an unpaired A-rich motif in the 5'-end of a target mRNA causing inhibition of the initiation of translation [17,18]. It is still not fully understood how Crc activity is regulated in different *Pseudomonas *species, nor whether a common unified regulatory system is employed. In *P. aeruginosa*, activity is regulated by small RNA, CrcZ, which has five A-rich motifs, that binds to the Crc protein and sequesters it [17]. Levels of the CrcZ sRNA, in turn, are regulated by a two-component system (CbrA/CbrB) and by RpoN. Interestingly, CbrAB and NtrBC form a network to control the C/N balance in both *P. aeruginosa *and *P. fluorescens *[19-21]. Furthermore, the presence of a readily available nitrogen source enhances the magnitude of CRC [22], two observations that are suggestive of a link between regulatory systems controlling C and N utilisation. Although the *crcZ *gene is present in other Pseudomonads, its role in regulating CRC outside of *P. aeruginosa *has not yet been demonstrated. Indeed, in *P. putida*, *crc *mRNA and Crc protein levels are higher under conditions where CRC is active, a phenomenon not observed in *P. aeruginosa*, suggesting that an alternative system of regulating CRC may be used in this species [23,24].

Much of what is known about CRC comes from work on mutants lacking the Crc protein in *P. aeruginosa *and *P. putida*. Initially, the key work in identifying the CRC system came from the isolation and characterisation of a *P. aeruginosa crc *mutant [25]. In this mutant, the succinate-mediated catabolite repression control (CRC) of glucose and mannitol transport and Entner-Doudoroff pathway enzymes was alleviated, thereby establishing the importance of Crc. More recently, the role of Crc has been examined on a global scale in *P. putida *[26] and *P. aeruginosa *[27] by carrying out transcriptome and proteome analyses of *crc *mutants. No less than 134 targets in *P. putida *and 65 targets in *P. aeruginosa *were differentially altered in expression in rich media as a result of a *crc *mutation. This indicates that *crc *is an important global regulator that superimposes an additional layer of regulation over many metabolic pathways that are otherwise regulated locally by specific regulatory elements that control only one or a few genes. The global analyses of the *P. putida *and *P. aeruginosa crc *mutants indicates that CRC is responsible for the hierarchical assimilation of amino acids from rich media, with pathways required for assimilation of valine, isoleucine, leucine, tyrosine, phenylalanine, threonine, glycine and serine inhibited by Crc [26,27]. Additionally, the *P. aeruginosa crc *mutation was shown to alter the expression of targets with roles in anaerobic respiration, antibiotic resistance and virulence [27]. Recent work on a *crc *mutant of *P. putida *DOT-T1E established that Crc is not involved in the induction of pathways for nutrient utilisation since the mutant grows on the same range of carbon and nitrogen sources as the wild type strain [28]. This is in contrast to the *E. coli *CCR system where the cAMP-CRP complex is responsible for the induction of genes for utilisation of less favoured carbon sources such as lactose [29].

The role of CRC in regulating linear and aromatic hydrocarbon utilisation pathways in *P. putida *has received a lot of attention because of the potential implications of CRC on bioremediation processes. The utilisation of alkanes and a wide range of aromatic compounds including benzene and toluene are subject to CRC in *P. putida *[16,30-34]. Indeed Crc mediated post-transcriptional control of the *pheA *and *pheB *toluene degradation genes [31], the *benR *activator of benzene degradation [33], the *alkS *activator of alkane degradation [16], the *xylR *activator of the TOL genes and *xylB *(benzyl alcohol dehydrogenase) [34] and the *bkdR *activator of branched-chain keto acid dehydrogenase [35] has been demonstrated. The Crc protein is known to bind *in vitro *to mRNAs of the P_U_, P_M_, P_S1_, P_R1_, promoters and mRNAs of the *xylMABN *(and weakly to *xylL *and *xylQ*) genes of the pWW0 toluene degradation plasmid in *P. putida *indicating that these targets may also be regulated by directly by Crc [34]. Besides the role that CRC plays in the bioremediation activities of *P. putida*, little else is known about the control that CRC imposes on the ecological functions of Pseudomonads other than for virulence-associated functions in *Pseudomonas aeruginosa*. A *crc *mutant of *P. aeruginosa *PA14 was defective in biofilm formation and type IV pilus-mediated twitching motility [36]. and a *crc *mutant of *P. aeruginosa *PAO1 displayed increased susceptibility to some antibiotics as well as defects in type III secretion, motility and expression of quorum sensing-regulated virulence factors [27]. Given the range of ecological functions that *Pseudomonas *may perform there is great scope for Crc to be a significant regulator beyond the realm of primary metabolism. For instance, glucose metabolism is subject to CRC and gluconate is a product of glucose metabolism. Gluconate itself is linked to phosphate solubilisation [9] and biocontrol [37] and there is a link between the ability to produce gluconate and the levels of antimicrobial compounds produced such as 2,4-diacetylphloroglucinol and pyoluteorin [38]. Additionally, recent evidence indicates that there is a link between primary metabolism and secondary metabolism controlled by the GacS/Rsm system [39]. This suggests that there is great potential for CRC to interact with other regulatory networks, at least indirectly, and it is therefore a high priority to better understand the Crc regulon.

Based on the size of the Crc product and the proposed mechanism of action, it is thought that Crc binding must occur within -70 to +16 bp relative to the origin of translation [18]. There remain, however, very few known direct targets of Crc: only *benR *[33], *alkS *[18], *xylR *and *xylB *[34] mRNAs from *P. putida *and *amiE *mRNA [17] (product of the amidase gene *amiE*), in *P. aeruginosa *have been demonstrated to bind Crc. To extend the number of direct targets known, we carried out a bioinformatic analysis using genome information from sequenced *Pseudomonas *strains. By identifying the specific targets in pathways that are known to be regulated by CRC, it will be possible to determine precisely how different Pseudomonads control nutrient uptake and utilisation. Furthermore, the analysis is expected to identify new pathways and processes, not previously known to be CRC-regulated. A better understanding of how *Pseudomonas *species use CRC will enhance knowledge of the ecology of these bacteria and will facilitate efforts to exploit the metabolic capacity of these bacteria in industrial and environmental microbiology.

## Results and discussion

### Crc binding site detection: estimation of the numbers of Crc regulated genes in *Pseudomonas*

**Figure 1:**
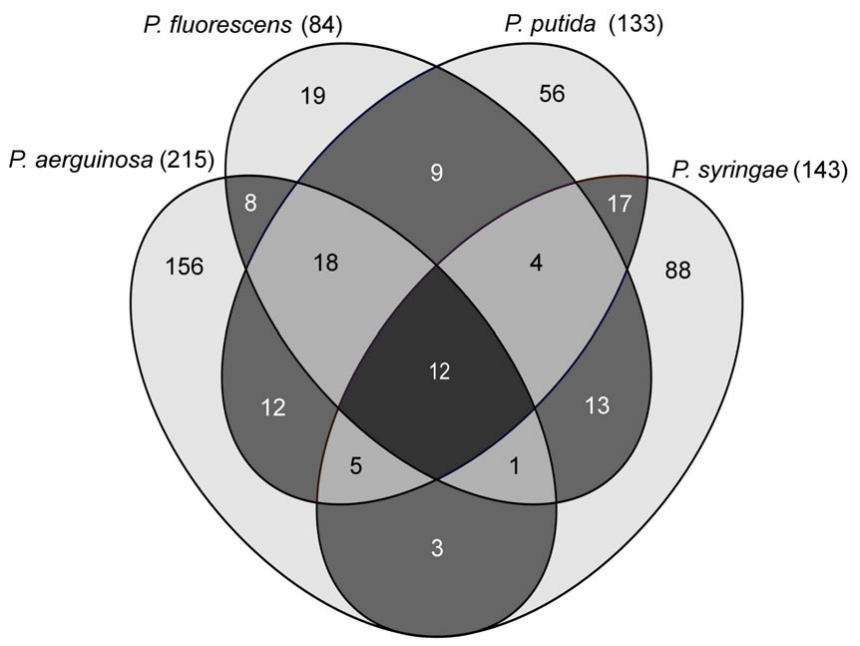
**Interspecific variations of the Crc regulon**. Venn diagram showing a four way comparison of Crc candidates in *P. aeruginosa*, *P. fluorescens*, *P. putida *and *P. syringae*. Numbers in parentheses correspond to total number of Crc candidates in each species.

Following the recent discovery that the Crc protein binds to an A-rich motif in the -70 to +16 region of target mRNAs [17,18] a search was performed to catalogue the occurrences of this motif in the forward orientation in the upstream regions of all protein-encoding genes in *Pseudomonas *strains present in the RSAT database [40] at the time of analysis. These were *P. aeruginosa *(PAO1, PA14, PA7 and LESB58), *P. fluorescens *(Pf0-1, Pf-5 and SBW25), *P. putida *(KT2440, F1 and W619) and *P. syringae *(B728a and DC3000). It was reasoned that if a gene is under direct Crc control, the binding site should be present in that gene in all representatives of a particular species. Accordingly, only genes with the A-rich motif (AAnAAnAA) in the upstream region of intraspecies orthologs for all strains of a given species were considered as candidates (Additional file 1). In total, 421 candidate genes were identified, with an estimated false discovery rate of 27% (see materials and methods). *P. aeruginosa *has the highest number (215) of Crc candidates, *P. syringae *and *P. putida *had 143 and 133, respectively while *P. fluorescens *has the lowest number (84) (Figure 1). This difference in the number of possible CRC-regulated genes is likely to be a consequence of the taxonomic organisation within the genus, in particular the diversity of *P. fluorescens *species. A consequence of this diversity is that the core genome of *P. fluorescens *is significantly smaller than that of *P. aeruginosa *and so the pool of orthologous genes that are potentially regulated by Crc is lower [41-45]. Twelve Crc candidates are common to all four *Pseudomonas *species while a further 28 Crc candidates are present in three out of the four species examined (Figure 1). Taken together, these 40 Crc candidates represent the predicted core Crc regulon of *Pseudomonas *(Table 1). Many of these Crc candidates are annotated as having roles in nutrient transport and metabolism, fitting with the idea of CRC as a means of controlling hierarchical assimilation of nutrients from the environment. Most putative Crc targets are not part of the core regulon and are confined to a single or two species. These include the three Crc target genes (*alkS*, *benR *of *P. putida *and *amiE *of *P. aeruginosa*) that have been experimentally shown to bind Crc in the 5' region of the mRNA [17,18,33]. No orthologues of *benR *or *amiE *were detected outside of *P. putida *or *P. aeruginosa *species, respectively, and so these are species-specific targets. The absence of *alkS *in our dataset is due to its location on a mobile element (the *P. putida *OCT plasmid) that is only present in some strains of *P. putida*. In summation, the *Pseudomonas *regulatory network controlled by Crc ranges from genes that are regulated at a genus-wide level, down to genes that may only be regulated in certain strains within a particular species.

**Table 1:** Predicted core Crc regulon of Pseudomonas.

Gene name	Function	PAO1	PA14	PA7	LESB58	Pf0-1	Pf-5	SBW25	KT2440	F1	W619	B728a	DC3000	
	**Amino acid transport and metabolism**													
*putP*	Sodium/proline symporter	0783	54150	4736	45601	0453	0496	0452	4946	4818	0522	NM	NM	
	Probable amino acid permease	0789	54040	4729	45531	4561	4906	1103	1059	1100	1089	NO	NO	
*phhA*	Phenylalanine-4-hydroxylase	0872	52990	4644	44441	1499	1611	4458	4490	1424	3779	NM	NM	Y
*gltI*	Probable binding protein component of ABC transporter	1342	46910	4043	38391	4535	4871	1139	1071	1112	1100	NM	NM	
	Probable sodium/alanine symporter	2252	35460	2989	30521	NM	NM	NM	0496	0530	0551	5052	5500	
	Probable glutamine synthetase	2040	38140	3247	32821	5408	5930	5849	5184	5091	0279	4868	5310	
	**Carbohydrate transport and metabolism**													
*mltE*	Probable binding protein component of ABC maltose/mannitol transporter	2338	34420	2925	29651	2640	3070	2745	NO	NO	2041	2440	2707	
*oprB*	Glucose/carbohydrate outer membrane porin precursor	3186	23030	1943	18821	4366	4613	4842	1019	1057	4205	1117	1296	Y
	Probable binding protein component of ABC sugar transporter	3190	22980	1939	18781	4370	4617	4846	1015	1053	4209	1113	1292	Y
*lldP*	L-lactate permease	4770	63080	5490	51551	0753	0817	5278	4735	4601	0696	NO	NO	
*fruA*	PTS fructose IIC component	NM	NM	NM	NM	0795	0861	0806	0795	0818	4398	0823	0956	Y
glpF	Glycerol uptake facilitator protein	NM	NM	NM	NM	4531	4867	1143	1076	1117	1105	3904	4167	
	**Energy production and conservation**													
*coxB*	Cytochrome c oxidase, subunit II	0105	01290	0180	01061	0079	0061	0058	0103	0119	0122	NO	NO	
*pntB*	Pyridine nucleotide transhydrogenase, beta subunit	0196	02470	0277	01971	0112	0113	0111	0155	0173	5072	NM	NO	
*acsA*	Acetyl-coenzyme A synthetase	0887	52800	4627	44291	4293	4522	4766	4487	1428	3776	3572	1825	
	Putative glycerate 2-kinase	1499	45030	3833	39131	1595	1698	1800	4300	1569	3625	NO	NO	
	Probable D-beta-hydroxybutyrate permease	2004	38580	3285	33191	3078	3575	2629	3074	2650	2717	NO	NO	
	Putative acetate transporter	3235	22340	1890	18321	1607	1711	1813	1742	3977	1292	3757	1623	
*cstA*	Probable carbon starvation protein	4606	60950	5246	49911	4883	5352	5333	4641	4503	0798	4273	4638	
*phaC1*	Poly(3-hydroxyalkanoic acid) synthase 1	5056	66820	5794	54461	0394	0434	0396	5003	4877	0461	NO	NO	
*phaF*	polyhydroxyalkanoate synthesis protein PhaF	5060	66875	5799	54501	NM	NM	NM	5007	4881	0457	0391	5147	
	**Alginate metabolism**													
*algP*	Alginate regulatory protein	5253	69370	5998	56471	NM	NM	NM	0194	0215	0263	0054	0136	
	**Lipid metabolism**													
*fadD2*	Long-chain-fatty-acid--CoA ligase	3300	21340	1824	17661	4354	4599	4830	4550	1339	3845	3836	4098	
*estA*	Esterase	5112	67510	5845	55021	NM	NM	NM	0418	0452	4784	4606	0569	
	**Polyamine metabolism**													
*aphA*	Acetylpolyamine aminohydrolase	1409	46230	3930	40041	5631	6145	6061	5340	5249	0133	NO	NO	
	**Xenobiotic degradation and transport**													
*pcaK*	Benzoate transport	NM	NM	NM	NM	1266	1316	1362	1376	4347	1016	2124	2340	
	**DNA replication, recombination and repair**													
*recA*	RecA protein	3617	17530	1523	14181	1175	1231	1189	1629	4088	4030	1378	4033	
*hupA*	HU family DNA-binding protein	5348	70600	6125	57431	5600	6102	6032	5313	5222	0160	NM	NM	Y
	**nucleotide transport and metabolism**													
	Probable transporter	1519	44800	3815	38911	1711	4364	4355	4284	1584	3610	NO	NO	
*xdhA*	Xanthine dehydrogenase	1524	44710	3809	38041	1797	1889	4592	4278	1590	3604	NM	NM	
	**Translation**													
*rplR*	50S ribosomal protein L18	4247	09010	0853	06811	5063	5566	5511	0470	0503	4733	4532	0642	
tufB	Elongation factor Tu	4277	08830	0835	06631	5081	5584	5529	0452	0485	4751	4550	0624	
	**Unknown function**													
	Hypothetical protein	0754	54540	4766	45891	NM	NM	NM	1418	4303	1058	3966	4232	Y
	Probable transporter	1507	44950	3824	39041	1701	4371	4364	4290	1578	3616	NO	NO	
	Probable major facilitator superfamily (MFS) transporter	3709	16410	1427	12731	3359	2486	2159	0057	0073	0076	NO	NO	
	Hypothetical protein	3923	13130	1184	10541	4708	5116	0923	0765	0792	4424	0991	1149	
	Probable ATP-binding component of ABC transporter	4461	57930	5034	48401	0858	0916	0883	0953	0992	4262	4146	4452	
	Hypothetical protein	4570	60480	5210	49531	4863	5332	5174	0685	0716	4500	NO	NO	
	Hypothetical protein	5052	66760	5789	54421	0398	0438	0400	5090	4963	0375	NM	NM	
	Phosphotransferase domain-containing protein	NM	NM	NM	NM	1487	1597	4880	4500	1412	3789	3586	1810	

Loci with an A-rich motif in the upstream region in all strains tested for at least three species are shown. The numbers under strain names indicate the locus id, according to Genbank annotation, of the locus with the A-rich motif in the upstream region. NO (no ortholog) indicates that no orthologous locus was found. NM (no motif) indicates that the orthologous locus did not have the A-rich motif in the upstream region. Y indicates that the locus is has increased transcript and/or protein levels in a *crc *mutant of *P. putida *KT2442 (a spontaneous rifampicin resistant mutant of KT2440) [26].

### Comparison of predicted Crc-regulated candidates to experimental datasets

Other studies have made use of *crc *mutant strains derived from *P. putida *KT2440 and *P. aeruginosa *PAO1 to experimentally identify possible targets of the CRC system. To assess the level of concordance between the bioinformatic and experimental approaches, the datasets were compared (Table 2 and Table 3). Recently, transcriptome and proteome comparisons of transcript and protein abundances of *P. putida *KT2442, a spontaneous rifampicin-resistant mutant of strain KT2440, and an isogenic *crc *mutant have been performed in rich media [26]. The transcriptome and proteome data sets identified 134 genes that were differentially altered in expression either at transcriptional or translational level in the *crc *mutant. We compared this list of 134 genes to the lists of genes identified in our bioinformatic analysis, with the results presented in table 2. The initial comparison was to the 133 candidate genes that were bioinformatically predicted to be the core Crc regulon of *P. putida *and then to ensure that possible positive matches were not overlooked, we extended the comparison to the longer list of 294 candidates identified in *P. putida *strain KT2440 (only targets present in all three *P. putida *strains were shown in additional file 1). 18 common targets between the predicted *P. putida *Crc regulon and the transcriptome/proteome data were identified, and another 5 possible targets are seen when the comparison is with the full KT2440 list of candidates.

**Table 2:** Comparison of predicted Crc regulon of P. putida with transcriptome and proteome data.

Gene name	***putida***^**a**^	**KT2440**^**b**^	Function	mRNA	Protein
	NO	PP_0267	outer membrane ferric siderophore receptor	nd	1.6
*fruR*	NM	PP_0792	FruR transcriptional regulator	nd	2.3
*fruA*	PP_0795	PP_0795	PTS fructose IIC component	2.1	nd
*gap-1*	PP_1009	PP_1009	glyceraldehyde-3-phosphate dehydrogenase, type I	2.7	3.3
	PP_1015	PP_1015	probable binding protein component of ABC sugar transporter	2.3	4.9
*oprB-1*	PP_1019	PP_1019	Glucose/carbohydrate outer membrane porin OprB precursor	3.5	2.9
	PP_1059	PP_1059	probable amino acid permease	6.4	nd
*aatJ*	PP_1071	PP_1071	probable binding protein component of ABC transporter	3.3	7.7
	NM	PP_1400	dicarboxylate MFS transporter	2.5	nd
*tctC*	PP_1418	PP_1418	hypothetical protein	1.6	3.4
*cspA-1*	PP_1522	PP_1522	cold shock protein CspA	1.9	3.5
*ansA*	PP_2453	PP_2453	L-asparaginase, type II	2.4	3.1
	PP_3123	PP_3123	3-oxoacid CoA-transferase subunit B	9.1	4.5
	NO	PP_3434	hypothetical protein	6.7	nd
	NM	PP_3530	conserved hypothetical protein	2.0	nd
	PP_3593	PP_3593	amino acid ABC transporter, periplasmic amino acid-binding protein	nd	6.3
*bkdA-1*	PP_4401	PP_4401	3-methyl-2-oxobutanoate dehydrogenase	3.2	1.6
*phhA*	PP_4490	PP_4490	phenylalanine-4-hydroxylase	2.8	1.9
	PP_4495	PP_4495	aromatic amino acid transport protein AroP2	2.6	nd
*hmgA*	PP_4621	PP_4621	homogentisate 1,2-dioxygenase	5.0	7.8
	PP_4636	PP_4636	acetyl-CoA acetyltransferase	3.6	2.3
*hupA*	PP_5313	PP_5313	probable DNA-binding protein	3.8	nd
*accC-2*	PP_5347	PP_5347	acetyl-CoA carboxylase subunit A	2.4	nd

Genes differentially regulated, based on transcriptome and proteome data, in rich media in a *crc *mutant of *P. putida *KT2442 [26] are cross referenced with (a) predicted Crc targets from three *P. putida *strains (KT2440, F1 and W619) and (b) with predicted Crc targets from *P. putida *KT2440 alone. Values of mRNA and protein indicate the relative levels of transcripts and protein in transcriptome and proteome analyses respectively [26]. NO (no ortholog) indicates that no orthologous loci were detected in either or both of *P. putida *F1 and W619. NM (no motif) indicates that no A-rich motif was detected in the upstream region of the orthologous loci in *P. putida *F1 and W619.

**Table 3:** Comparison of predicted Crc regulon of P. aeruginosa with proteome data.

Gene name	PAO1	Function	protein
	PA0534	conserved hypothetical protein	2.03
*hpd*	PA0865	4-hydroxyphenylpyruvate dioxygenase	4.71
*oprD*	PA0958	Basic amino acid, basic peptide and imipenem outer membrane porin OprD precursor	1.75
	PA1069	hypothetical protein	4.28
	PA2553^a^	probable acyl-CoA thiolase	1.59
	PA2555	probable AMP-binding enzyme	1.54
	PA2776	conserved hypothetical protein	1.71
	PA3187^b^	probable ATP-binding component of ABC transporter	10.28
*edd*	PA3194	phosphogluconate dehydratase	2.17
	PA4500	probable binding protein component of ABC transporter	3.48
	PA4502^c^	probable binding protein component of ABC transporter	3.35
	PA4506^c^	probable ATP-binding component of ABC dipeptide transporter	8.43
*dadA*	PA5304	D-amino acid dehydrogenase, small subunit	2.36

Genes differentially regulated, based on proteome data, in rich media in a *crc *mutant of *P. aeruginosa *PAO1 [27] are cross referenced with predicted targets from all *P. aeruginosa *strains considered in this study. Values of protein indicate relative levels of protein in the *crc *mutant relative to levels in the wild type strain. Some genes are proximal to, and possibly in operons with, bioinformatically predicted Crc targets: (a) PA2553 is proximal to PA2555, (b) PA3187 is proximal to PA3186 and (c) PA4502 and PA4506 are proximal to PA4501.

A proteomic analysis comparing the wild type strain *P. aeruginosa *PAO1 to an isogenic *crc *mutant in LB broth was also recently performed [27]. Under these conditions, 46 proteins were present at higher levels in the *crc *mutant compared to the wild type strain, suggesting that these targets are negatively regulated by the CRC system. Comparing those 46 experimentally-identified targets with the 215 predicted Crc targets identified in our bioinformatic study, it is seen that 13 of the 46 targets overlap (Table 3). Of these, 9 common targets have a predicted Crc binding site in the gene itself and a further 4 targets are in operons downstream of predicted Crc targets (Table 3). When the comparison is expanded to include all 279 candidates identified in PAO1 no new matches were found. The authors of that study identified putative Crc-binding sites in the 5' region of 23 of the 46 genes, and suggested that these may be subject to direct Crc mediated regulation [27]. The criteria applied for identifying putative Crc-binding sites was less strict than our study (with respect to consensus and distance from AUG codon), which explains the difference between the 13 binding sites we propose and the 23 postulated by these authors.

The fact that 18/23 overlaps are in the core *P. putida *regulon (and a further 2 are only excluded because orthologues are absent) and that no new overlaps with experimental data are introduced when the predicted Crc-regulon of *P. aeruginosa *is considered reinforces the validity of using the presence of the motif in orthologous genes within a species as a selection criterion in the global bioinformatic screen. Although the overlap between the experimental and bioinformatic datasets appears low for *P. putida *- 18(23)/267 genes - this should not be entirely unexpected. Genes predicted by the bioinformatics but not identified experimentally could simply be because they were below experimental detection limits, or more likely because the growth conditions used favoured some classes of genes. Of course, some hits may represent false positives, and our analysis predicted that there are rates of 18% and 26% false positive hits for *P. aeruginosa *and *P. putida *respectively. These are also possible explanations for differences between our data set and the PAO1 proteome data despite the higher level of overlap between our data with PAO1 (13/46) than between our data with KT2440. It is interesting that all three studies identify amino acid metabolism as an important component of the Crc-regulon. This reflects Crc metabolic adaptations in a nutrient rich environment (which was the experimental condition) where various amino acids are the major carbon sources. Performing the transcriptome/proteome experiments under different growth conditions, would be likely to yield a different set of genes. Conversely, there were also targets identified in the experimental studies that did not feature in the bioinformatic analysis. The most likely explanation for this is that these are indirect rather than direct targets of Crc as they lack the predicted Crc binding site. It is also possible, however, that the strict criteria used in the bioinformatic analysis excluded some genuine targets, or that Crc has alternative or additional binding sites, perhaps used only under certain conditions. From comparing all the data, we can already see that this was probably the case with the *bkdA1 *gene, which was identified as a target experimentally in both *P. putida *and *P. aeruginosa*, but bioinformatically only in *P. putida *(Table 2). The proposed Crc binding site in *P. aeruginosa *is AACAAGAGAAACAA [27], which differs in some positions to the consensus AAnAAnAA used in the bioinformatic analysis. Ultimately, protein-mRNA binding studies will be needed to resolve all these Crc-binding possibilities.

### Crc regulates carbohydrate and amino acid utilisation

**Figure 2:**
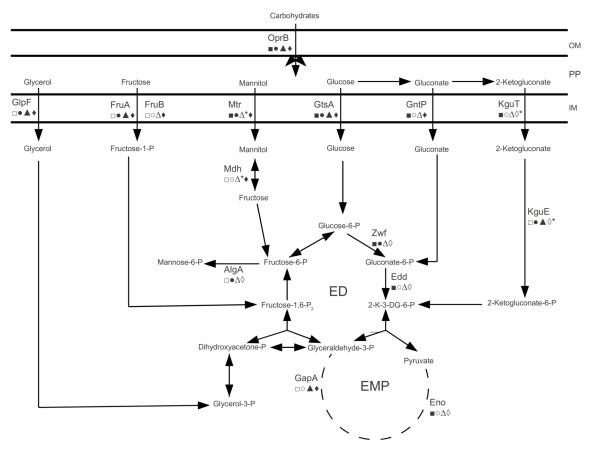
**Predicted Crc regulon of carbohydrate metabolism in *Pseudomonas***. Selected genes involved in carbohydrate transport and metabolism are shown along with their status *vis a vis *(predicted) Crc regulation. Genes from *P. aeruginosa *(squares), *P. fluorescens *(circles), *P. putida *(triangles) and *P. syringae *(diamonds) are shown, with filled/unfilled symbols indicating that the target in that species is/is not predicted to be regulated by Crc. An asterisk (*) after a symbol indicates where an orthologous locus is absent in the relevant species. OM - outer membrane; PP - periplasm; IM - inner membrane; ED - Entner-Doudoroff pathway; EMP - Embden-Meyerhoff pathway; 2-K-3-DG-6-P - 2-keto-3-deoxygluconate-6-phosphate. OprB - carbohydrate porin B; GlpF - glycerol transporter; FruAB - fructose phosphotransferase system; Mtr - mannitol transporter subunit; GtsA - glucose transporter subunit; GntP - gluconate transporter; KguT - 2-ketogluconate transporter; Mdh - mannitol dehydrogenase; AlgA - mannose-6-P isomerase; Zwf - glucose-6-P dehydrogenase; Edd - gluconate-6-P dehydratase; KguE - xylose isomerase; GapA - glyceraldehyde-3-P dehydrogenase; Eno - phosphopyruvate hydratase. Some steps of the Embden-Meyerhoff pathway are abbreviated with a dashed line for clarity.

In order to find a common pattern of Crc regulation in *Pseudomonas *spp., we examined the function associated with the Crc candidates. In Pseudomonads, intermediates of the TCA cycle such as succinate or citrate cause catabolic repression of pathways involved in metabolism of carbohydrates, amino acids and other carbon sources [14,46]. Therefore, it is not surprising to find predicted Crc targets involved in such pathways. Indeed, our analysis highlights six interspecies Crc candidates involved in carbohydrate metabolism (Table 1). Since these candidates are all related to transport, it is tempting to speculate that Crc is responsible for direct down-regulation of transporters of carbohydrate utilisation, which would result in the indirect down-regulation of the relevant catabolic enzymes due to the lack of inducing molecules in the cytoplasm. Closer inspection of the intra-species Crc candidates, however, shows that some genes linked to carbohydrate metabolism could also be directly regulated by Crc (Additional file 1). For example, in *P. aeruginosa *and *P. fluorescens *species, the gene, *zwf*, encoding glucose-6-phosphate dehydrogenase has a Crc motif, whereas in *P. putida *and *P. syringae *species, the gene, *gap-1*, encoding glyceraldehyde-3-phosphate dehydrogenase has a Crc motif. When viewed in an integrated way, it is seen that there are two distinct patterns to the regulation of genes in this class (Figure 2). When present, sugar transporters are generally subject to CRC control, whereas the regulation of downstream sugar metabolism is species-specific with respect to genes encoding catabolic enzymes. Interestingly, the same trend is observed for amino acid metabolism where most of the interspecies Crc candidates are involved in transport (Table 1), whereas intraspecies candidates are involved in metabolism (Additional file 1).

It is notable that another gene, *cstA*, with a predicted role in carbon starvation stress alleviation was also implicated as a Crc candidate. The CstA protein is involved in peptide transport that would assist the cell in escaping carbon starvation [47]. In *Escherichia coli*, induction of the *cstA *gene depends on cAMP and Crp [48] indicating that this locus is subject to CCR in *E. coli*. It is intriguing that a locus is implicated as being subject to catabolite repression in both *Escherichia *and *Pseudomonas*, despite differences in preferred nutrient sources and mechanisms of catabolite repression.

### Crc regulates transcriptional activators that are induced during stationary phase

Crc also seems to regulate proteins involved in transcriptional regulation, as previously described [33]. Indeed the gene, *hupA*, encoding a bacterial histone like protein (HU-like protein), possesses a Crc motif in the *P. aeruginosa*, *P. putida *and *P. fluorescens *species. HU proteins are ubiquitous DNA binding factors that are involved in the structural maintenance of the bacterial chromosome and other events that require DNA binding [49]. In contrast to the structurally related integration host factor (IHF), HU proteins bind DNA in a sequence-independent manner. Generally, *Pseudomonas *possesses five HU/IHF copies per genome [50]. Two of these ORFs encode the two subunits of the IHF (integration host factor) protein (*ihfA *and *ihfB*), whereas *hupA *(or *hupP*), *hupB *and *hupN *encode HU-like proteins. Although the precise role of *hupA *is not known, HU-like proteins are required for transcription from the σ^54^-dependent *Ps *promoter of the toluene degradation pathway in *P. putida *[51], which is known to be subject to control by the CRC system. Identification of the Crc motif would be consistent with the idea that Crc impacts indirectly on the transcription level of a subset of genes through translational regulation of the regulatory genes *hupA *or *ihfB*. This may also explain some of the indirect targets of Crc identified in the transcriptome/proteome analysis discussed earlier [26]. The expression of *hupA*, *hupB *and *hupN *has been monitored during *P. putida *KT2440 growth [52]. Interestingly, whereas *hupB *and *hupN *transcript abundances are maximal in exponential phase, *hupA *expression seems to be activated during stationary phase. Remarkably, another Crc candidate of *P. aeruginosa *and *P. syringae*, *ihfB*, has increased expression during transition of cells from exponential growth to stationary phase [53]. This observation is not an isolated phenomenon as other predicted Crc targets, for example *cstA *[47,48] and polyhydroxyalkanoate biosynthesis (*phaC1 *or *phaZ*) [54], are also induced at the onset of stationary phase. CRC is depressed during stationary phase [24] so these observations on expression are consistent with a role for Crc in repressing expression of target genes during active growth.

### Crc regulates virulence-related traits

It was shown previously that a *crc *mutant of *P. aeruginosa *PA14 was defective for biofilm formation and type IV pilus-mediated twitching motility [36] and a *crc *mutant of *P. aeruginosa *PAO1 is compromised in type III secretion, motility, expression of quorum sensing-regulated virulence factors and was less virulent in a *Dictyostelium discoideum *model [27]. Therefore, we searched for bioinformatic evidence that Crc integrates nutritional status cues with the regulation of virulence-related traits. We postulate that Crc might regulate some steps in alginate biosynthesis in *Pseudomonas*. Alginate production is linked to the conversion of microcolonies from a non-mucoid to a mucoid phenotype. In *P. aeruginosa *this phenotype marks the transition to a more persistent state during pulmonary infection, characterised by antibiotic resistance and accelerated pulmonary decline [55]. The regulation of alginate production in *Pseudomonas *is highly complex and involves the interaction of many regulatory systems [56]. In this study, the transcriptional activator AlgP, involved in the transcription of a key alginate biosynthetic gene, *algD *[57] encoding GDP-mannose 6-dehydrogenase, is predicted, to be directly regulated by Crc in *P. aeruginosa, P. putida *and *P. syringae *species. In this case, the interspecific Crc regulation blocks the synthesis of a transcriptional regulator which leads to indirect regulation of the biosynthetic pathway, reminiscent of the cases of *alkS *and *benR *in *P. putida *[18]. Nevertheless, at the species level, Crc is also predicted to regulate some enzymes directly. In *P. aeruginosa*, Crc also is predicted to bind to *alg8 *and *algF *transcripts which encode a subunit of alginate polymerase [58,59] and an alginate acetylation protein [60] respectively. The synthesis of the alginate precursor, mannose-6-phosphate, encoded by *algA*, is predicted to be under the control of Crc in *P. fluorescens *only (Figure 2). The additional levels of regulation of alginate in *P. aeruginosa*, could reflect the importance of this exopolysaccharide for persistence in specialised ecological niches, including inside the host.

Another interesting Crc target is *estA *encoding an autotransporter protein with esterase activity [61] that is indispensable for rhamnolipid production [62]. Rhamnolipids are surface-active molecules that play a role in biofilm fluidity [63] and are toxic against a variety of microorganisms [64]. Preliminary experiments confirm that rhamnolipid production is a Crc-regulated trait in *P. aeruginosa *(data not shown). Moreover, inactivation of the *estA *gene in *P. aeruginosa *also influenced other virulence-related functions like swimming, twitching and swarming in a rhamnolipid-independent fashion [62]. Rhamnolipids have numerous features in common with polyhydroxyalkanoic acid (PHAs), a metabolic storage material involved in bacterial stress-resistance and biofilm formation [65]. Firstly they are both synthesised in response to the presence of excess carbon where other nutrients, such as nitrogen or phosphorus, are growth limiting [54,64,66]. Secondly, both molecules are composed of 3-hydroxydecanoic acids connected by ester bonds. Interestingly, *phaC1 *[67] and *phaF *[68] encoding a PHA polymerase and PHA transcriptional regulator respectively are also predicted to be Crc regulated in *P. aeruginosa, P. putida *and *P. syringae *species. Notwithstanding the role of PHA in attachment of *P. aeruginosa *to surfaces [65], the implication of Crc in PHA production may also be interesting from an industrial point of view since it is hoped that PHA accumulating bacteria may be exploited in bioplastic production [69]. Under high carbon:nitrogen ratios, PHA and rhamnolipids are produced and represent carbon sinks to accommodate an inability to metabolise an excess of carbon over nitrogen. One possible function of the CRC system is to integrate C/N metabolism by regulating the production of carbon sink compounds such as PHA and rhamnolipid. This could be mediated by the CbrAB/NtrBC links outlined earlier.

## Conclusions

CRC is an important global control network employed by *Pseudomonas *to optimise growth with available nutrients in a variety of environments. This analysis aimed to predict the set of targets that are directly regulated by the Crc protein in four species of *Pseudomonas*. As expected, genes involved in the metabolism of less favoured nutrients were identified. An interesting feature, however, was that the regulation of transporters is a conserved feature of Crc regulation in *Pseudomonas spp*. while the regulation of particular enzymatic steps and transcriptional activators is generally present in a more species-dependent manner. This suggests that different *Pseudomonas *species have fine-tuned CRC to reflect the ecology of that particular species. In addition to anticipated effects on sugar metabolism, there are indications from the data that Crc may play a role in maintaining the carbon/nitrogen balance in *Pseudomonas *and this is worthy of further study. It was postulated that identifying Crc targets might enhance knowledge of some applied aspects of *Pseudomonas *and one example of this was the prediction that Crc regulates steps in polyhydroxyalkanoate (PHA) synthesis in *P. putida*, as this is of interest for the production of biodegradable bioplastics. In the case of *P. aeruginosa*, the analysis revealed that alginate production and other traits linked to virulence may be under CRC control. It was especially intriguing to discover that Crc may play a role in regulation of globally important DNA binding proteins such as HU and IHF and thus regulate, indirectly, many pathways that depend on the DNA bending properties of these proteins for transcription or repression. These novel aspects of Crc regulation therefore deserve further investigation given the potential that it may enhance our understanding of the integration of nutritional status cues with the regulation of important activities of the *Pseudomonas*.

## Methods

Positions -70 to +16 relative to the origin of translation of all protein encoding genes of available *Pseudomonas spp*. were downloaded from the regulatory sequence analysis tool (RSAT) [40] using the retrieve sequence function. Genes containing an A-rich (AAnAAnAA) motif in the -70 to +16 region were identified using a script in Perl. Translated protein encoding sequences were downloaded from the *Pseudomonas *genome database [70] and used to create local blast databases with formatdb [71]. Orthologous genes were identified as best hits using blastp analysis (blastall v2.2.22) [71,72] against local databases. Cut-offs of 50% identity over at least 80% of the sequence length and an expected value (e-value) of 1e-10 were applied. Orthology was confirmed by reciprocating the blastp analysis. Since the A-rich motif is short and degenerate it is expected that occurrences of the A-rich motif that are unrelated to Crc binding will be detected in this analysis, giving rise to false positive hits. In order to estimate the rate of false positive hits in our analysis we searched for the A-rich motif in the reverse orientation of the upstream regions of orthologous loci [73]. Since the A-rich motif in the reverse orientation is unrelated to Crc binding it is reasoned that this estimates the rate of occurrence of the A-rich motif in the sequence fragments tested. Predictably it was found that the use of more strains per species resulted in lower estimated rates of false positives (*P. aeruginosa *- 4 strains, 18% estimated false positives; *P. fluorescens *- 3 strains, 32% estimated false positives; *P. putida *- 3 strains, 26% estimated false positives; *P. syringae *- 2 strains, 41% estimated false positives). Thus, it is estimated, based on the weighted mean false discovery rate, that approximately 73% of the Crc candidates in additional file 1 are genuine targets for Crc binding. Functional information about the translated protein sequences was obtained from the sequence headers and by performing Blast2GO analysis [74].

## Authors' contributions

PB, JPM and FOG conceived the study. PB performed the bioinformatic analyses, PB and MB interpreted the data and JPM and FOG oversaw the study. PB and MB prepared figures, tables and additional files presenting the data and PB, MB, JPM and FOG drafted the manuscript. All authors read and approved the final manuscript.

## Supplementary Material

Additional file 1 **Crc candidates identified in every *Pseudomonas *spp**. List of every locus bearing a Crc motif in *P. aeruginosa*, *P. fluorescens*, *P. putida *and *P. syringae *species. The numbers under strain names on the left indicate the locus id, according to Genbank annotation, of the locus with the A-rich motif in the upstream region. The numbers under the strain names on the right indicate the position of the A-rich motif relative to the origin of translation. Click here for file
